# Approach to the Evaluation and Management of Wide Complex Tachycardias

**Published:** 2002-10-01

**Authors:** Patrick Lam, Samir Saba

**Affiliations:** Division of Cardiac Electrophysiology, University of Pittsburgh, Pittsburgh, PA

## Introduction

Wide complex tachycardia (WCT) refers to a cardiac rhythm of more than 100 beats per minute with a QRS duration of 120 ms or more on the surface electrocardiogram (ECG). It often presents a diagnostic dilemma for the physician particularly in determining its site of origin, which can be ventricular or supraventricular. In one series, only 32% of clinicians correctly diagnosed ventricular tachycardia (VT) in patients who presented with WCT [1]. Prompt diagnosis of the etiology of WCT is, however, essential since immediate care is frequently required. Diagnostic and therapeutic errors can produce poor outcome especially when ventricular tachycardia is not recognized [2,3].

WCT that is grossly irregular typically represents atrial fibrillation with aberrant conduction or preexcitation. If its rate exceeds 200 beats per minute, the likelihood of atrial fibrillation with conduction over an accessory pathway should be entertained. In this article, we will discuss the approach to the evaluation and management of regular WCT.

## Differential Diagnosis

The differential diagnosis of regular wide complex tachycardia includes three major categories: ventricular tachycardia (VT), supraventricular tachycardia (SVT) with aberrance and preexcited tachycardia.

Ventricular tachycardia is the most common etiology accounting for about 80 % of WCT [2-4]. Ventricular tachycardia originates from the ventricles and does not require any supraventricular tissues for its maintenance. A significant number of patients with VT are hemodynamically stable and are asymptomatic or present with palpitation or mild lightheadedness [5]. This adds to the dilemma of diagnosing WCT since there is a misconception amongst physicians that hemodynamic stability is not consistent with a diagnosis of VT. The failure to recognize hemodynamically stable VT can often lead to inappropriate therapy and dangerous sequelae.

SVT with aberrance accounts for a small portion of WCT. SVT typically originates in atrial tissue and/or AV junction and utilizes the normal atrioventricular (AV) conduction system for ventricular activation. Aberrance occurs when there is delay or block in the His-Purkinje system during antegrade conduction of impulses over the normal AV fascicles. Essentially, all types of SVT with aberrant conduction can present as a WCT. These include atrial tachycardias, atrioventricular nodal reentrant tachycardias, orthodromic reciprocating tachycardias, and other rare forms of SVT.

Preexcited tachycardias are conducted antegradely over an accessory pathway (AP). Evidence for the presence of an AP can be manifest of the surface electrocardiogram (ECG) which can show intermittent or continuous presence of a delta wave associated with a short PR interval. The delta wave represents the part of the ventricular myocardium that is depolarized through the AP.

An increasingly recognized cause of WCT is electrographic artifact [6]. The clinical consequences of misinterpreting artifact as WCT are significant [7] and can subject patients to a wide range of unnecessary diagnostic and therapeutic procedures including cardiac catherization and implantation of cardiac devices.

Ventricular paced rhythms represent a small portion of WCT. Sometimes the bipolar ventricular spike is small and difficult to see. The WCT is typically of LBBB pattern and superior leftward axis with right ventricular apical pacing. This pattern can vary significantly with the site of pacing. The tachycardia can be due to any form of SVT where the pacemaker is tracking sensed atrial activity and is pacing the ventricles at a fast rate. It can also represent a pacemaker-mediated tachycardia where the pacemaker is tracking retrogradely conducted atrial activity during ventricular pacing. A history and physical examination revealing the presence of a cardiac implantable device provide important clues to this diagnosis.

## Clinical Approach to the Differential Diagnosis

### History

A history of structural heart disease, particularly of coronary artery disease or prior myocardial infarction, strongly suggests a diagnosis of VT in patients presenting with WCT [8,9]. The overall physical appearance of the patient, his or her presenting symptoms and hemodynamic measurements are generally not very useful [1,2]. A history of medications taken by the patient, particularly those that are known to have proarrhythmic effects, such as most anti-arrhythmic drugs, can also help with the discrimination between the various possible etiologies of WCT.

### Physical Examination

Although the blood pressure and heart rate are not particularly useful in differentiating WCT of ventricular versus supraventricular origin, certain physical signs such as the demonstration of AV dissociation strongly suggest a diagnosis of VT. These signs include the presence of cannon A waves on jugular vein examination, irregular intensity of the first heart sound by auscultation, and varying systolic blood pressure. These physical signs are highly specific and sensitive for the diagnosis of VT [10]. Vagal maneuvers such as carotid sinus massage as well as the use of AV nodal blocking agents such as adenosine may cause transient ventriculoatrial block leading to AV dissociation that establishes the diagnosis of VT [11]. Although the termination of WCT by physical maneuvers such as carotid sinus massage and Valsalva are strongly suggestive of SVT, there are well documented reports of similar occurrences in patients with VT [12,13]. Termination of WCT by adenosine, digoxin, calcium-channel blockers, or b-blockers is again strongly suggestive of a diagnosis of SVT. There are however well documented cases of similar responses with VT [14,15]. Of utmost importance however is the avoidance by physicians of medications such as verapamil that can compromise the hemodynamic stability of patients with VT. This can lead to the abrupt clinical deterioration of an otherwise stable patient and can lead to hazardous consequences [16].

An uncommon but sometimes useful semi-invasive procedure in the evaluation of patients with WCT is the placement of a transesophageal probe to record the atrial electrical activity and establish the diagnosis [17].

### The Electrocardiogram (Figure 1, Figure 2)

The ECG remains a cornerstone in the diagnosis of WCT. A plethora of different diagnostic criteria have been suggested to distinguish between the various etiologies of WCT, each with different sensitivity and specificity [18-21].

The most useful ECG criterion in establishing the diagnosis of VT is the presence of AV dissociation with more ventricular than atrial events. This practically rules out the possibility of the WCT representing an SVT. Fusion beats during WCT also imply the presence of AV dissociation, since they are the result of the simultaneous activation of the ventricular myocardium through both the normal conduction system and from an ectopic ventricular focus. A capture beat, also known as Dressler beat, is a narrow QRS complex resulting from a well-timed P wave capturing the ventricular myocardium through the normal conduction system during VT. A narrow complex beat, however, can also occur during WCT in patients with SVT and underlying bundle branch block (BBB). In this case, a ventricular premature beat originating from or close to the non-conducting bundle branch fuses with the impulses traveling down the contralateral bundle resulting in simultaneous ventricular activation on both sides of the septum and in a narrow QRS complex.

The QRS duration has been proposed as a criterion to differentiate between WCT of ventricular and supraventricular origins. Wellens and colleagues found that 69% of VTs had QRS duration greater than 140 ms, whereas no SVT had QRS duration in this range [20]. This criterion was further extrapolated to patients with underlying BBB [2]. Ventricular tachycardia was the likely diagnosis when the QRS duration was greater than 140 ms in patients with right BBB QRS patterns and greater than 160 ms in those with left BBB QRS patterns.

The cardiac electrical axis during WCT is also useful to determine the origin of the abnormal rhythm. In the presence of a right superior (northwest) axis, the WCT is unlikely to represent an SVT, since this ECG pattern is inconsistent with any type of typical bundle-branch or fascicular block. Also, electrical concordance across the precordium (all QRS complexes pointing in the same direction in ECG leads V1 through V6) is rarely consistent with an SVT, and typically represents an anteroapical (negative concordance) or a posterobasal (positive concordance) left ventricular VT.

Morphology is also often used to differentiate between WCT of ventricular from supraventricular origins [19]. In the presence of a right BBB QRS pattern in lead V1, the diagnosis is likely to be VT if the left QRS peak (R) is taller than the right peak (r') or if the QRS complex is biphasic with an Rs or qR pattern. In the presence of a left BBB QRS pattern, VT is the likely diagnosis if in lead V1, the duration of the initial r wave is greater than 30 ms, the time from the beginning of the QRS to the nadir of the S wave is greater than 70 ms, and if the down stroke of the S wave is notched. The morphology of the QRS complex in lead V6 is also used to discriminate between SVT and VT. If in lead V6 the QRS complex has a monophasic QS or biphasic rS morphology with an r to S ratio < 1 during right BBB WCT, then VT is likely. If the intrinsicoid deflection in V6 is greater than 80 ms, then VT is also more likely. All these criteria have been clinically evaluated. Their usefulness in the distinction between SVT and VT during WCT is limited and not as good as was initially suggested by early reports [22]. 

## Role of Invasive Electrophysiologic Testing

In certain situations, patient can have episodes of WCT that may require invasive electrophysiologic (EP) testing to make a diagnosis. The primary indication for EP testing in patients with WCT is if the correct diagnosis is unclear after analysis of available ECG tracings and the knowledge of the correct diagnosis is necessary for patient care. This is listed by the American College of Cardiology/American Heart Association task force as a class I indication for performing an EP study [23]. Class I indications are conditions for which there is evidence and/or general agreement that a given procedure or treatment is useful and effective.

Most patients do not have spontaneous episodes of tachycardia during EP testing and have to be induced into the abnormal rhythm by burst pacing or premature stimulation at various sites and cycle lengths, with and without β-adrenergic stimulation. It is important however to make sure that the arrhythmia induced is the clinical arrhythmia responsible for the patient's presentation. This is achieved by careful comparison of the morphology and rate of the induced tachycardia with those of prior recordings obtained by ECG, holter monitors, or event monitors. Complete electrophysiologic testing is usually necessary not only to differentiate between VT and SVT but also to distinguish between the various forms of these arrhythmias.

As mentioned before, AV dissociation is the hallmark of ventricular tachycardia. The difficulty arises when patients have retrograde 1:1 VA activation during VT. This can be difficult to distinguish from an SVT. In this situation, recording a His bundle electrogram and analyzing its relationship to the atrial and ventricular electrograms is essential for establishing the diagnosis. Also, performing electrophysiologic and pharmacologic maneuvers to try to dissociate the atria from the ventricles can be of great help.

## Management of Wide Complex Tachycardia

The initial approach to the management of the patient who presents with WCT depends primarily on the patient's hemodynamic status. Patients with low blood pressure, pulmonary edema, severe angina, or other evidence of poor perfusion associated with the WCT should be promptly cardioverted back into normal rhythm, using synchronized electrical direct current. Hitting the patient on the chest, also called "thump version" can sometimes terminate a WCT presumably by mechanically inducing a premature ventricular complex that interrupts the reentrant circuit of the tachycardia.

For the hemodynamically stable patient, time should be spent on analyzing data from the history, physical examination, and ECG recordings to reach a diagnosis with a high degree of confidence. As mentioned above, from a statistical standpoint, the most likely diagnosis in patients with WCT is VT. Assuming this diagnosis even in the hemodynamically stable patient until proven otherwise is the safest approach to management.

Because it can terminate both SVT and VT, Amiodarone (class III) is the agent of choice for stable WCT particularly if the etiology is uncertain. It is also particularly useful in patients with poor ejection fraction, since it has a favorable hemodynamic profile [24]. Procainamide (Class IA) can also be used in the initial treatment of hemodynamically stable WCT. Its main disadvantage is that it cannot be administrated rapidly secondary to hypotension. Lidocaine (IB) is very useful for VT of ischemic origin. It does not terminate SVT, however, and can result in confusion at high doses. Adenosine is a useful diagnostic and therapeutic medication when a diagnosis of SVT is suspected. It works by blocking conduction through the AV node, which is an essential part of the tachycardia circuit in most SVTs. It can at times terminate automatic atrial tachycardias and rare adenosine-sensitive VTs. Because of its very short half-life, it rarely causes complications in patients with WCT. An exception to this general rule is in the patient who presents with irregular WCT due to atrial fibrillation and preexcitation over a manifest accessory pathway. In this instance, adenosine can block conduction through the AV node and favor conduction of atrial fibrillation wavelets down the bypass tract, possibly leading to ventricular fibrillation.

After the acute management of an episode of WCT, long-term plans should be made to prevent the recurrence of the episodes, minimize their symptomatic impact, and protect the patient against sudden cardiac death. In general, the therapeutic options include pharmacological treatment with anti-arrhythmic drugs, device treatment with the automatic implantable cardioverter-defibrillator, and catheter ablation procedures. A combination of two or more of these modalities is sometimes necessary.

In the patient with SVT, a catheter-based approach to therapy is curative in the overwhelming majority of patients and provides protection against sudden cardiac death in patients with the Wolf-Parkinson-White syndrome who have a rapidly conducting antegrade accessory pathway. In those patients whose ablation procedure fails or who choose not to undergo an invasive test, numerous antiarrhythmic medications can be used to suppress the SVT including b-blockers, calcium channel blockers, or class I and class III anti-arrhythmic drugs.

In patients with poor ejection fraction and sustained ventricular tachycardia, device therapy is usually recommended. Pharmacologic therapy is used as an adjunctive therapy to minimize the episodes of VT and prevent defibrillator shocks. Occasionally, catheter ablation is needed as an adjunctive therapy for frequent or incessant VTs that fail to respond to medications.

Not all VTs, however, are life-threatening and require device therapy. Idiopathic VTs occur in structurally normal hearts and carry a very good prognosis. These include right and left ventricular outflow tract VTs and left ventricular fascicular (verapamil-sensitive) VTs. Like SVTs, idiopathic VTs can also be cured with catheter ablation with good success rates [25,26].

## Summary

Patients who present with WCT constitute a challenge to the diagnostic acumen of physicians. If misdiagnosed, these patients can be mismanaged, which can lead to lethal consequences. In the hemodynamically unstable patient, synchronized electrical cardioversion is the initial therapy. In the stable patient, physicians should use all clues provided by the history, physical examination, and ECG, in reaching the correct diagnosis. Until proven otherwise, any WCT should be managed as if it were VT, in keeping with the consideration of "First, do no harm". This implies that physicians should refrain from using long acting AV nodal blocking agents that can induce hypotension in an otherwise stable patient.

## Figures and Tables

**Figure 1:**
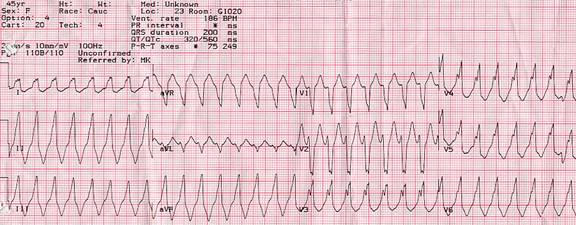
Wide complex tachycardia in a 45 year old woman with a structurally normal heart. This rhythm was incidentally noted on a cardiac monitor during an elective surgery. The patient had a similar episode 4 years earlier in the context of another elective surgery. She was completely asymptomatic during this rhythm. Note the left bundle, right axis morphology of the QRS complex. The width of the QRS complex is 200 milliseconds. The initial r wave in V1 is about 35 milliseconds in duration. There is a notch on the down stroke of the S wave in V1. All these morphologic features point to a diagnosis of VT. There is no precordial concordance in ECG leads V1 through V6 however, a feature more frequently seen in SVTs. Adenosine (12 mg IV) failed to terminate the tachycardia or change the rhythm. This WCT represents an idiopathic VT originating from the right ventricular outflow tract. Paper speed is 25 mm/s.

**Figure 2:**
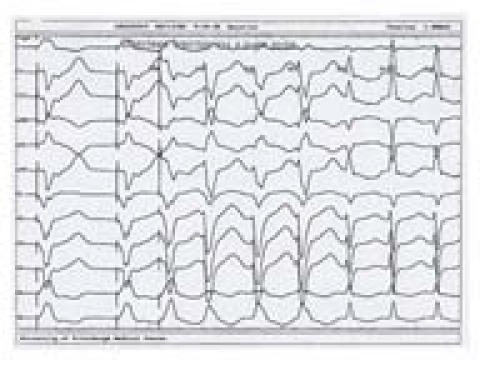
WCT due to an SVT with left BBB aberrance. Note that the tachycardia is induced by ventricular stimulation with a single premature beat delivered at the right ventricular apex. After the third beat of tachycardia, the rhythm changed from WCT with left BBB morphology to a narrow complex tachycardia. On EP testing, this tachycardia was found to represent an orthodromic atrioventricular reciprocating tachycardia using a concealed left atrioventricular free-wall accessory pathway for its retrograde limb. The accessory pathway was successfully ablated, and the tachycardia could no longer be induced. The clue to the diagnosis of SVT with aberrance in this case is the presence of both narrow and wide complex tachycardias at nearly the same cycle lengths in the same patient. Paper speed is 75 mm/s.
